# From Data Acquisition to Data Fusion: A Comprehensive Review and a Roadmap for the Identification of Activities of Daily Living Using Mobile Devices

**DOI:** 10.3390/s16020184

**Published:** 2016-02-02

**Authors:** Ivan Miguel Pires, Nuno M. Garcia, Nuno Pombo, Francisco Flórez-Revuelta

**Affiliations:** 1 Instituto de Telecomunicações, University of Beira Interior, 6201-001 Covilhã, Portugal; ngarcia@it.ubi.pt or ngarcia@di.ubi.pt (N.M.G.); ngpombo@di.ubi.pt or ngpombo@ubi.pt (N.P.); 2 Altranportugal, 1990-096 Lisbon, Portugal; 3 ALLab - Assisted Living Computing and Telecommunications Laboratory, Department of Informatics, University of Beira Interior, 6201-001 Covilhã, Portugal; 4 ECATI, Universidade Lusófona de Humanidades e Tecnologias, 1749-024 Lisbon, Portugal; 5 Faculty of Science, Engineering and Computing, Kingston University, Kingston upon Thames KT1 2EE, UK; F.Florez@kingston.ac.uk

**Keywords:** sensor data fusion, accelerometer, data collection, signal processing, sensors signal, activities of daily living

## Abstract

This paper focuses on the research on the state of the art for sensor fusion techniques, applied to the sensors embedded in mobile devices, as a means to help identify the mobile device user’s daily activities. Sensor data fusion techniques are used to consolidate the data collected from several sensors, increasing the reliability of the algorithms for the identification of the different activities. However, mobile devices have several constraints, e.g., low memory, low battery life and low processing power, and some data fusion techniques are not suited to this scenario. The main purpose of this paper is to present an overview of the state of the art to identify examples of sensor data fusion techniques that can be applied to the sensors available in mobile devices aiming to identify activities of daily living (ADLs).

## 1. Introduction

Sensors are present in much of the equipment used in everyday life by everyone, including mobile devices, which are currently applied in ambient assisted living (AAL) systems, such as smartphones, smartwatches, smart wristbands, tablets and medical sensors. In these devices, sensors are commonly used to improve and support peoples’ activities or experiences. There are a variety of sensors that allow the acquisition of the various types of data, which can then be used for different types of tasks. While sensors may be classified according to the type of data they manage and their application purposes, the data acquisition is a task that is highly dependent on the user’s environment and application purpose.

The main objective of this paper is to present a comprehensive review of sensor data fusion techniques that may be employed with off-the-shelf mobile devices for the recognition of ADLs. We present a classification of the sensors available in mobile devices and review multi-sensor devices and data fusion techniques.

The identification of ADLs using sensors available in off-the-shelf mobile devices is one of the most interesting goals for AAL solutions, as this can be used for the monitoring and learning of a user’s lifestyle. Focusing on off-the-shelf mobile devices, these solutions may improve the user’s quality of life and health, achieving behavioural changes, such as to reduce smoking or control other addictive habits. This paper does not comprehend the identification of ADLs in personal health or well-being, as this application ecosystem is far wider and deserves a more focused research, therefore being addressed in future work.

AAL has been an important area for research and development due to population ageing and to the need to solve societal and economic problems that arise with an ageing society. Among other areas, AAL systems employ technologies for supporting personal health and social care solutions. These systems mainly focus on elderly people and persons with some type of disability to improve their quality of life and manage the degree of independent living [1,2]. The pervasive use of mobile devices that incorporate different sensors, allowing the acquisition of data related to physiological processes, makes these devices a common choice as AAL systems, not only because the mobile devices can combine data captured with their sensors with personal information, such as, e.g., the user’s texting habits or browsing history, but also with other information, such as the user’s location and environment. These data may be processed either in the device or sent to a server using communication technologies for later processing [3], requiring a high level of quality of service (QoS) to be needed to achieve interoperability, usability, accuracy and security [2]. The concept of AAL also includes the use of sophisticated intelligent sensor networks combined with ubiquitous computing applications with new concepts, products and services for P4-medicine (preventive, participatory, predictive and personalized). Holzinger *et al*. [4] present a new approach using big data to work from smart health towards the smart hospital concept, with the goal of providing support to health assistants to facilitate a healthier life, wellness and wellbeing for the overall population.

Sensors are classified into several categories, taking into account different criteria, which include the environmental analysis and the type of data acquired. The number and type of sensors available in an off-the-shelf mobile device are limited due to a number of factors, which include the reduced processing capacity and battery life, size and form design and placement of the mobile device during the data acquisition. The number and type of available sensors depend on the selected mobile platform, with variants imposed by the manufacturer, operating system and model. Furthermore, the number and type of sensors available are different between Android platforms [5] and iOS platforms [6].

Off-the-shelf mobile devices may include an accelerometer, a magnetometer, an ambient air temperature sensor, a pressure sensor, a light sensor (e.g., photometer), a humidity sensor, an air pressure sensor (e.g., hygrometer and barometer), a Global Positioning System (GPS) receiver, a gravity sensor, a gyroscope, a fingerprint sensor, a rotational sensor, an orientation sensor, a microphone, a digital camera and a proximity sensor. These sensors may be organized into different categories, which we present in Section 2, defining a new classification of these sensors. Jointly with this classification, the suitability of the use of these sensors in mobile systems for the recognition of ADLs is also evaluated.

**Figure 1:**
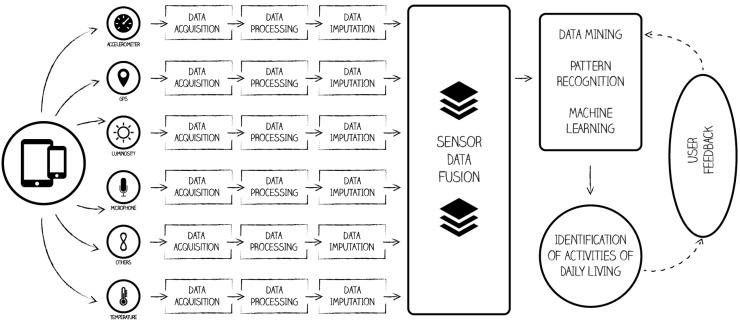
Schema of a multi-sensor mobile system to recognize activities of daily living.

The recognition of ADLs includes a number of different stages, namely data acquisition, data processing, data imputation, sensor data fusion and data mining, which consist of the application of machine learning or pattern recognition techniques (Figure 1).

As shown in Figure 1, the process for recognizing ADLs is executed at different stages, which starts with the data acquisition by the sensors. Afterwards, the data processing is executed, which includes the validation and/or correction of the acquired data. When data acquisition fails, data correction procedures should be performed. The correction of the data consists of the estimation of the missing or inconsistent values with sensor data imputation techniques. Valid data may be sent to the data fusion module, which consolidates the data collected from several of the available sensors. After the data fusion, ADLs can be identified using several methods, such as data mining, pattern recognition or machine learning techniques. Eventually, the system may require the user’s validation or feedback, either at an initial stage, or randomly in time, and this validation may be used to improve, to train and to fine-tune the algorithms that handle the consolidated data.

Due to the processing, memory and battery constraints of mobile devices, the selection of the algorithms has to be done in a particular way, or otherwise, the inappropriate use of resource-greedy algorithms will render the solution unusable and non-adoptable by the users.

The remaining sections of this paper are organized as follows: Section 2 proposes a new classification for the sensors embedded in off-the-shelf mobile devices and identifies different techniques related to data acquisition, data processing and data imputation; Section 3 is focused on the review of data fusion techniques for off-the-shelf mobile devices; some applications of mobile sensor fusion techniques are presented in Section 4; in Section 5, the conclusions of this review are presented.

## 2. Sensors

Sensors are hardware components that have the capability to capture different types of signals. Sensors are widely available in equipment used daily, including in smartphones, smartwatches, tablets and specific devices, including medical and industrial devices, and may be used to collect data in a plethora of situations. Examples of the use of sensors’ data include measuring some property of the sensor’s environment, such as chemical sensing, the measurement of motion, touch and proximity data or acoustic or imaging detection.

### 2.1. Sensor Classification

In AAL systems, sensors are mainly used to measure data from the user and his/her environment. For example, the sensors available in most mobile devices allow the detection of movements and activities of the user, which may lead to identifying the ADLs, with a high accuracy.

The research about sensor data fusion techniques should include the identification of the different types of sensors available for use. The analysis of the different types of sensors is commonly named sensor classification, consisting of the definition of different classes to which sensors may be assigned. Examples of features that define the different sensor classes include the purpose of the sensor, its working environment and the type of data they acquired [7,8]. The environment itself can be classified as controlled, uncontrolled, static, dynamic, uncertain and undefined [7,8]. The classification of sensors presented in this paper has a special focus on the identification of the ADLs.

According to [9], the sensors used for sleep detection include the measurement of different activities and physical phenomena, such as electro-encephalography (EEG), electro-cardiography (ECG), blood pressure, photoplethysmography, non-cardiac electropotentials, oxygen saturation, respiration, body movement, arterial tonometry and temperature. These measurements include the use of different sensors, such as accelerometer, gyroscope, heart band, microphones and video cameras, to assess the body’s acceleration and its vital signs while recording audio and video.

For the detection of physical activities, the authors in [10] propose the use of inertial sensors, *i.e.*, accelerometers and gyroscopes, complemented with orientation measurement using magnetic sensors, e.g., a compass and a magnetometer, and location measurement using location sensors, e.g., a GPS receiver. However, location measurement can also be achieved using inertial sensors and acoustic sensors, e.g., a microphone [11].

Sensors can be used to perform the measurement of physical activities in controlled or uncontrolled environments. When used in controlled environments, these sensors, e.g., the video sensors, may be distributed in different places to measure the movements in a defined and often a static spatial area [12]. Gentili and Mirchandani [13] make use of counting sensors, image sensors and automatic vehicle identification (AVI) readers to identify the origin and the location of an object. The use of static or dynamic images relies heavily on image processing algorithms to extract the relevant features. Due to the large variety of data that can be extracted from still images or videos, it is an autonomous research topic. On the other hand, Lee *et al*. [14] make use of a wearable electrogoniometer composed of a knee angular and a three-axis accelerometer sensor to detect the ADLs in uncontrolled environments, presenting also four different machine learning techniques for detecting occurrences of walking. Other studies were performed in uncontrolled environments using different mobile technologies. The accuracy of the results is influenced by several constraints also presented in this paper.

Sensors may be grouped into different categories related to the identification of the movements and ADLs. One of the usual categories is related to acoustic sensors, which can be used in almost all environmental scenarios. This category includes simple microphones, silicon microphones [15] and other acoustic wave devices [16], which are usually used for physical sound wave sensing.

Another proposed category integrates chemical sensors, especially useful for detecting the presence of specific molecules in the air and/or the environment. In [17], the classification of these sensors is presented as metal-oxide, semiconductive polymers, conductive electroactive polymers, optical, surface acoustic wave and electrochemical gas sensors. These types of sensors are also used for medical purposes, e.g., for the detection of the correct dosage in a patient’s treatment [18].

Another class of sensors includes mechanical sensors, such as mass sensors, strain sensors [19], pressure sensors, contact sensors, mechanical switches and others. These sensors may be used to detect movements of the user’s body.

Magnetic sensors may be used to complement the sensing of the different movements. These include search-coil magnetometers, fluxgate magnetometers, superconductor magnetometers, hall effect sensors, magnetoresistive magnetometers, spin-valve transistors, giant magnetoimpedance magnetic sensors, magnetodiodes, magnetotransistors, magnetostrictive magnetometers, magneto-optical sensors and micro-electro-mechanical systems (MEMS)-based magnetometers [20].

Optical sensors may also be used to measure the parameters of ADLs [21], which include photoplethysmography, fiber optic sensors, infrared sensors and radio frequency sensors. These sensors are able to work with adverse temperatures, corrosion/erosion surroundings, high-vibration, voltage and pressure environments [22], but these are not usual on smartphones.

The detection of physical activities can also be achieved by sensors used in medical applications. The majority of these sensors can be included in the categories previously mentioned, such as inertial sensors, e.g., accelerometers, gyroscopes, inclinometers or pedometers, mechanical sensors, e.g., ball/tilt/foot switches, sole switches and force-sensitive resistors, acoustic sensors, e.g., microphones, optical sensors, e.g., infrared sensors, radio frequency sensors, e.g., radio frequency identifiers (RFID), New Field Communication (NFC) and Ubisense Real-Time Location Systems (RTLS), atmospheric sensors, e.g., barometers and hygrometers, electrical sensors, e.g., electromyogram (EMG), ECG, electrodermal activity (EDA) and electrooculography (EOG) sensors, magnetic sensors, e.g., magnetometers, photoelectric sensors, e.g., oximeters, and chemical sensors, e.g., actinometers [23].

Another category of sensors includes the location sensors, such as GPS receiver or WiFi location methods. Zhang *et al*. [24] presented a comparison between the accuracy of the use of WiFi location and the use of a GPS receiver, in which the GPS signal is limited in indoor environments, and in this case, the use of a WiFi location method is advised.

In [25], wearable sensors are considered. These include the sensors embedded in mobile devices, such as the GPS receiver, accelerometer, proximity sensor, camera, microphone, gyroscope, light sensor, gravity sensor and other sensors connected by, for example, Bluetooth (e.g., heart rate sensors, chemical sensors and others) [26]. Wearable devices of this type are mainly used in the medical area to capture data that will be used for diagnosis [27,28], e.g., EEG, optical sensors, thermal sensors, acoustic sensors, magnetic sensors or mechanical sensors.

A variety of sensors is commonly available in off-the-shelf mobile devices, improving the ability to detect some physiological parameters anywhere and at anytime. Taking previous research into consideration and having as a goal the selection of sensors that may be used in off-the-shelf devices to allow the identification of ADLs, a new classification of sensors is proposed with the following categories: magnetic/mechanical sensors, environmental sensors, motion sensors, imaging/video sensors, proximity sensors, acoustic sensors, medical sensors, chemical sensors and force sensors.

Table 1 presents a non-exhaustive list for the sensors embedded in mobile devices and external sensors for each category. In addition, other sensors may be connected to the off-the-shelf mobile devices using over-the-air technologies, improving the capabilities of the data collection, but as these are not integrated into the mobile device itself, they are not considered in this review.

**Table 1:** Sensors classified by categories.

Category	External Sensors	Mobile Sensors
Magnetic/Mechanical sensors	Compass; Magnetometer; Strain sensors; Search-coil magnetometer; Fluxgate magnetometer; Superconductor magnetometers; Hall effect sensor; Magnetoresistive magnetometers; Spin-valve transistors; Giant magnetoimpedance magnetic sensors; Magnetodiode; Magnetotransistor; Magnetostrictive magnetometers; Magneto-optical sensor; MEMS Based Magnetometers; Ball/tilt/foot switch; Sole pressure switch; Pressure sensors; Contact sensors; Mechanical switches	Magnetometer; Compass
Environmental sensors	Barometer; Humidity; Light sensor; Thermal sensor	Ambient air temperature and pressure; Light Sensor; Humidity Barometer; Photometer; Thermal sensor
Location sensors	GPS receiver; Automatic Vehicle Identification (AVI) readers; Ubisense Real-Time Location Systems (RTLS); Wi-Fi Location-Based Services	GPS receiver; Wi-Fi Location-Based Services
Motion sensors	Accelerometer; Gyroscope; Pressure sensor; Gravity sensor; Inclinometer; Pedometer; Rotational sensor	Accelerometer; Gravity sensor; Gyroscope; Rotational vector sensor; Orientation sensor
Imaging/Video sensors	Digital camera; 3D camera; Optical sensor; Infrared sensor	Digital camera; Infrared sensor
Proximity sensors	Proximity sensor; Touch sensor; RFID; Tactile sensor; NFC	Proximity sensor; Touch sensor; RFID; NFC
Acoustic sensors	Microphone; Silicon microphones; Acoustic wave devices; Surface acoustic wave	Microphone
Medical sensors	EEG; ECG; EMG; EOG; EDA; Photoplethysmogram; Blood pressure and arterial tonometry; Respiration; Dosage control/detection; Stress sensors; Heart rate sensors; electrooculography; electrodermal activity sensors	None
Chemical sensors	Oxygen saturation; Aroma sensors; Metal-oxide; Semi conductive polymers; Conductive electro active polymers; Electrochemical gas sensors; Actinometer	None
Optical sensors	Photoplethysmography sensors; Fiber optic sensors; Infrared sensors; Radio frequency sensors	Infrared sensors; Radio frequency sensors
Force sensors	Force sensitive resistor; Mass sensor; Fingerprint sensor	Fingerprint sensor
Photoelectric sensors	Oximeter	None

### 2.2. Data Acquisition

Data acquisition depends on the particular characteristics of the sensors selected to perform the recognition of ADLs, on the environments where data are captured and also on the architecture of each system. The data acquisition process is accomplished by a module embedded in the mobile device and consists of the measurement and conversion of the electrical signals received by each sensor into a readable format [29].

Several challenges are associated with the data acquisition process when recognizing ADLs, including the positioning of the mobile device, the actual data sampling rate and the number of sensors to be managed [30]. The problems associated with data acquisition in an off-the-shelf mobile device influence the correct extraction of meaningful features of the ADLs. As the sensors are embedded in the mobile device, they cannot be located separately in different parts of the body; rather, the mobile device needs to be situated in an usual and comfortable position. Another issue related to mobile devices is the power consumption of the data acquisition tasks. Therefore, the limited battery capacity constraint needs to be taken into account to ensure a successful continuous operation of mobile applications [31] by developing and applying lightweight acquisition methods.

The main advantage of using mobile devices for data acquisition is related to the possibility to acquire data anywhere and at anytime [32]. However, it has some limitations in the performance of the data acquisition for real-time applications. Multitasking execution patterns differ among mobile devices, because these depend on their processing ability, memory and power capabilities, which are very limited, and on the operating system and on the number and type of mobile applications currently installed and/or running.

The *Acquisition Cost-Aware QUery Adaptation (ACQUA)* framework, presented in [31], consists of a query processing engine implemented in an off-the-shelf mobile device that dynamically modifies both the order and the segments of data streams requested from different data sources (sensors). The *ACQUA* framework starts by learning the selectivity properties of several sensor streams and then utilizes such estimated selectivity values to modify the sequence in which the mobile device acquires data from the sensors. The authors said that it is supported by some basic automated storage and retrieval system (ASRS) algorithms for acquisition cost-aware query processing. Query specification can be made using ASRS query evaluation algorithms, the disjunctive normal form or optimizing for multiple predicates on the same stream. The main features of *ACQUA* are the accommodation of the heterogeneity in sensors’ data rates, the determination of the packet sizes and radio characteristics, the adaptation of the dynamic changes in query selective properties and the support of multiple queries and heterogeneous time window semantics. The authors claim that it can result in a 70% reduction in the energy overhead of continuous query processing, by reducing the volume of sensor data that is sent over-the-air to a server between a mobile device and its attached sensors, without affecting the fidelity of the processing logic [33].

Lim *et al*. [31] reviewed different frameworks to optimize data acquisition. The *Orchestrator framework* focuses on resource-sharing between multiple context-aware applications executing queries independently [34,35]. The *ErdOS framework* views sensors available in off-the-shelf mobile devices as a shared resource and seeks to rationally distribute the consumption for all resources [36]. The *LittleRock prototype* uses a special low-energy coprocessor to decrease the computational energy spent in embedded processing of on-board sensor data streams [37]. The *Jigsaw continuous sensing engine* implements a pipelined stream processing architecture that adaptively triggers different sensors at different sampling rates to fit the context accuracy required by different applications [38]. The *SociableSense framework* combines the cloud-based computation and adaptive sensor sampling to reduce the computational and sensing overheads during continuous mobile sensing [39]. The *BBQ approach* builds a multi-dimensional Gaussian probability density function of the sensors’ likely data values and then uses conditional probabilities to determine, in an iterative manner, the next sensor whose value is most likely to resolve a given query [40]. These frameworks try to improve data acquisition and support the preparation of data for their later processing.

Table 2 presents a summary of the data acquisition frameworks, including their features and limitations. The data can be acquired by different methods, and the correct definition of the position of the sensors is the main factor that influences the data acquisition process. The definition of the best positioning for the sensors improves the reliability of the data acquisition methods implemented by the mobile operating systems, improving the data acquisition and data processing techniques. The selection of the best data acquisition methods depends on the purpose of use, the type of data acquired and their environment [41,42,43,44].

**Table 2:** Examples of data acquisition methods. *ACQUA*, *Acquisition Cost-Aware QUery Adaptation*; ASRS, automated storage and retrieval system.

Method	Features	Limitations
*ACQUA* framework	It is a query processing engine implemented on an off-the-shelf mobile device that dynamically modifies both the order and the segments of data streams requested from different data sources; supported by some basic ASRS algorithms for acquisition-cost-aware query processing; seeks to additionally reduce the communication energy overheads involved in acquiring the data wirelessly from additional external sensors; it is complementary to the *Jigsaw* and *LittleRock* frameworks.	It does not exploit correlations, which means that it lacks the predictive power of representations based on probabilistic models.
*Orchestrator framework*	Used for resource-sharing between multiple context-aware applications executing queries independently; enables the platform to host multiple applications stably, exploiting its full resource capacity in a holistic manner.	Timing relations are not known.
*ErdOS framework*	Distributes the consumption for all resources of the off-the-shelf mobile device; restarts the jobs in case of failure.	Statically configured and non-extensible; difficult to adapt for each case.
*LittleRock prototype*	Uses a special low-energy coprocessor to decrease the computational energy spent in embedded processing of on-board sensor data streams; loads the interactions with sensors and gives the phone’s main processor and associated circuitry more time to go into sleep mode; flexible storage.	Limited resources of the mobile devices.
*Jigsaw*	Balances the performance needs of the application and the resource demands of continuous sensing on the phone; comprises a set of sensing pipelines for the accelerometer, microphone and GPS sensors.	Robustness inferences.
*SociableSense framework*	Combines the cloud-based computation and adaptive sensor sampling to reduce the computational and sensing overheads during continuous mobile sensing.	Limited resources of the mobile devices.
*BBQ approach*	Builds a multi-dimensional Gaussian probability density function of the sensors’ likely data values and then uses conditional probabilities to determine, in an iterative manner, the next sensor whose value is most likely to resolve a given query; similar to *ACQUA*.	Similar to *ACQUA*.

### 2.3. Data Processing

Data processing is the next step of a multi-sensor mobile system used to process the sensor data. Data processing is a complex process, which also depends on environmental conditions, the types of sensor, the capabilities of the mobile device used and the types of data collected. The type of application also influences the use of data processing methods. Data processing may be executed locally, using the capabilities of the mobile device, or at the server side, sending the collected data to a remote server, where the data processing methods are executed [42,45,46]. For server-side processing, the mobile device is only required to acquire the sensors data and to present the results to the user. As frequent sensor sampling operations and further data processing can significantly reduce the battery lifetime and the capacities of the mobile device [47,48], several methods may be used to process different types of data. For example, while for audio processing, the time-scale modification (TSM) algorithm and, for medical imaging, remote processing methods are highly recommended, for other sensors, data can be processed locally in the mobile device without significant consumption of local resources [49,50,51]. Data processing methods may include a segmentation method, which divides a larger data stream into smaller chunks appropriate for processing, and a definition of the window size [52].

According to Pejovic and Musolesi [53], the challenges of data processing in mobile devices lie in the adaptation, context-driven operation, computation, storage and communication. The adaptation and the context-driven operation have several solutions that include the adaptive sampling, the hierarchical modality switching, the harnessing domain structure and the use of cloud offloading. Possible solutions for computation, storage and communication are hierarchical processing, cloud offloading and hardware co-processing.

Several systems and frameworks to classify and to process sensors’ data have been developed during the last few years. An option is, after contexts are extracted from the collected data, to discard part of the data to reduce memory usage [54]. Methods such as support vector machines (SVM), artificial neural networks (ANN), Bayes classifiers and k-nearest neighbour (KNN) algorithms, among others, may be used for data processing. In several research studies, the analysis and processing of sensor data include complex tasks that need high processing and memory capabilities [55]. In such cases, these activities need to be performed at the server-side.

In [54], Imai *et al*. present a rule-based data processing engine for sensor nodes to minimize the amount of energy needed to process the tasks. The data processing may be performed on a computer located in the neighbourhood of the sensor nodes, which processes the data and provides the results, obtaining a good balance between reducing the network load and advanced sensor data analysis and processing.

Yamada *et al*. [56] present a location-based information delivery method using *StreamSpinner*, which achieves efficient stream data processing based on novel multiple continuous query optimization techniques.

In [57], the Dandelion system is presented, achieving good results with *senselet*, a smartphone-style, platform-agnostic programming abstraction for in-sensor data processing. Dandelion provides a unified programming model for heterogeneous systems that span diverse execution contexts, supporting data-parallel applications.

Dolui *et al*. [58] defined two architectures: the Device Data Processing Architecture and the Server Data Processing Architecture. The Device Data Processing Architecture is designed to acquire the data from the sensors embedded in an off-the-shelf mobile device and to process the data locally. This architecture is useful when the processing methods require low resources, such as processing the accelerometer data, proximity sensor data and others. On the contrary, the Server Data Processing Architecture consists of the dispatch of the data collected to a remote server allowing the computation of a large amount of data, as well as computations of a complex nature. This architecture is employed for instance with data acquired with the GPS receiver and the imaging sensors.

Imai *et al*. [59] defined a method for data processing using the similarity of motions between observed persons. The method presented implements sensor data processing using a neighbour host, executed in two phases: a basic action phase and a changing sensor node settings phase [59]. In the basic action phase, a neighbouring host receives sensor data from the sensor nodes. Then, this host analyses and processes the data and sends the results to a different host. In the changing sensor node settings phase, when analytic results of sensor data fulfill the conditions determined in advance, the neighbouring host instructs sensor nodes to change the settings. Next, the sensor data processing method based on similarity is defined, where the system acquires and accumulates a newly-observed person’s acceleration data, while it estimates the related motions using a similar observed person’s profile. When the system acquires sufficient acceleration data of a newly-observed person, the system creates a profile that is added to the knowledge base.

The *ACQUA framework* [31,33] optimizes the data processing, using ASRS algorithms. The ACQUA framework can also be implemented in systems with a remote processing architecture or in systems that process the tasks locally.

Reilent *et al*. [41] developed an open software architecture for patient monitoring, which supports semantic data processing and (soft) real-time reasoning. Usually, in medical environments, the context awareness and decision making are performed in a remote server, which returns the results to the mobile device [42]. Another option used for processing the collected data is the use of a cloud-based server that processes the data in real time. However, it makes the mobile device and data processing dependent on a constant Internet connection [60].

In order to avoid this constraint, some tele-medicine systems have implemented data processing locally on the mobile device. For instance, Postolache *et al*. [61] implemented advanced data processing, data management, human computing interfacing and data communication using a smartphone running the Android operating system.

Augmented reality is also an application relevant for AAL. Paucher and Turk [32] implemented a system for image processing that used a remote server. The system implements a nearest neighbour search of descriptors using a k-dimensional (KD)-Tree structure, applying it for each image. Other algorithms for image processing using the camera of a mobile device are presented in [62].

Table 3 summarizes the different architectures for data processing, presenting the most relevant methods and achievements. It is often described in the literature that the data that can be processed locally on the mobile device include the motion or positioning sensors. However, the processing of images or videos is computationally expensive, and due to the low memory and processing capabilities of mobile devices, it is highly recommended that these tasks use external processing.

**Table 3:** Data processing: architectures and methods.

Architectures	Methods	Achievements
Device Data Processing Architecture	Dandelion system; SVM; ANN; Bayes classifiers; KNN algorithms; location-based information delivery method using *StreamSpinner*	Acquisition of the data from the sensors embedded in an off-the-shelf mobile device; process the data locally; the results are rapidly presented to the user; processing methods should require low resources; using segmentation methods, a larger data stream is divided into smaller chunks improving the methods; the correct definition of the window size is important for achieve good results.
Server Data Processing Architecture	SVM; ANN; Bayes classifiers; KNN algorithms; nearest neighbour search of descriptors using a KD-Tree structure.	Dispatching of the data collected to a remote server allowing the computation of a large amount of data, as well as computations of complex nature; in some cases, the data processing may be performed on a computer located in the neighbourhood of the sensor nodes; in server-side processing, the mobile device and data processing are dependent on a constant Internet connection.

### 2.4. Data Imputation

Data acquisition with sensors embedded in off-the-shelf mobile devices for real-time recognition of ADLs may fail in many circumstances. Firstly, the acquisition process fails when the sensors report unexpected values. Secondly, due to the multitasking nature of many of the mobile devices, this can happen anytime and for any given collection scenario.

Depending on the timing of the missing data included in a subset of sensors’ data, the missing data are classified as missing completely at random (MCAR) when the missing values are randomly distributed by all time instants [63,64]. The missing data are classified as missing at random (MAR) when the missing values are randomly distributed by subsets of the collected data [63,64]. Additionally, the missing data are classified as missing not at random (MNAR) when the missing values are not randomly distributed [63,64].

Several methods to minimize the effects of missing data have been developed, estimating the missing values based either on other values correctly obtained or on external factors. In [63], the Imputation Tree (ITree) method is presented, which is a tree-based algorithm for missing values imputation. This method constructs a missing pattern tree (MPT), which is a binary classification tree for identifying the absence of each observation. It uses clustering techniques, e.g., K-means clustering, to impute missing values and linear regression analysis to improve data imputation.

In [65], a multi-matrices factorization model (MMF) for the missing sensor data estimation problem is formulated, which uses statistical methods to estimate the missing values. There are other methods for sensor data imputation that employ KNN-based imputation [66], multiple imputation [67], hot/cold imputation [68], maximum likelihood and Bayesian estimation [69] and expectation maximization [70]. In general, these methods are used to verify and increase the consistency of sensor data.

The KNN method and its variants, such as KNNimpute (K-nearest neighbour imputation), SKNNimpute (sequential K-nearest neighbour method-based imputation) and MKNNimpute (K-nearest neighbour imputation method based on Mahalanobis distance), are the most used methods [71]. Other cluster-based imputation methods exists, such as KMI (K-means-based imputation) [72] and FCMimpute (fuzzy C-means clustering imputation) [73].

For audio signal imputation purposes, Smaragdis *et al*. [74] used nearest neighbours and singular value decomposition (SVD) algorithms. The SVD algorithm is executed after nearest neighbour clustering, replacing all of the missing values with an initial value, computing the SVD of the resulting matrix and replacing the missing values with their prediction according to the SVD decomposition, repeating the process until the change in the imputed missing data falls below some user-defined threshold.

In [75], a generalized trend diffusion (GTD) method is used to create small datasets. It applies the so-called shadow data and membership functions for increasing the knowledge when using back propagate-based (BP) neural networks to predict the missing values.

In [76], random recursive partitioning (RRP) is used to generate a proximity matrix used in non-parametric matching problems, such as hot-deck missing data imputation and average treatment effect estimation. In [77], a discrete model is presented to improve data imputation at the time of collection, reducing the errors and repairing the data if possible.

Other approaches [64] have also been studied to improve data imputation, such as ignoring and deleting data (e.g., listwise deletion and discarding instances), available-case methods (e.g., pairwise deletion), non-model based imputation procedures (e.g., unconditional mean imputation, conditional mean imputation) and model-based imputation procedures. Other implicit and explicit models exist for data imputation. The implicit models are based on implicit assumptions on the proximity between individuals belonging to the dataset (e.g., hot deck imputation, cold deck imputation, substitution method, composite methods), and the explicit models are based on a statistical model to describe the predictive distribution of missing data (e.g., linear regression, logistic regression, multiple imputation methods, the expectation-maximization (EM) algorithm, probabilistic neural networks, fuzzy min–max neural networks, general regression auto associative neural network and distribution free methods, such as non-parametric regression and tree-based methods).

The use of big data brings other challenges in data processing that consist of the extraction of relevant information to enable the correct analysis, discovery and interpretation of the data. Pombo *et al*. [55] presented a predictive model using the radial basis function neural network (RBFNN) combined with a filtering technique aiming at the estimation of the electrocardiogram (ECG) waveform, which supports healthcare professionals on clinical decisions and practices. The study [55] is related to the PhD thesis, presented in [78], that starts with the analysis of several methods for machine learning prediction and finalizes with the creation of a clinical decision support system. The methods studied are the rule-based algorithms (RBA), the ANN, the rough and fuzzy sets (RFS) and the statistical learning algorithms (SLA).

The data imputation methods can be applied in off-the-shelf mobile devices with some technical limitations, due to the capacities of these devices. For instance, in [79], neural networks are used to perform data imputation for a visual system.

Table 4 presents a summary about the data imputation methods analysed on this paper, presenting the types of data acquired and achievements obtained.

**Table 4:** Examples of data imputation methods.

Types of Data	Models	Achievements
MCAR	listwise deletion; pairwise deletion; ITree method; KNN method and their variants; KMI; FCMimpute; SVD; GTD method; BP neural networks; RRP; MMF; MPT; hot/cold imputation; expectation maximization; Bayesian estimation; unconditional mean imputation; conditional mean imputation; ANN.	These methods improve the identification of the absence of each observation; the use of clustering techniques and linear regression analysis improves the data imputation; the data imputation also increases the consistency of the data; other improvements of data imputation are ignoring and deleting the unusable data; another measured variable can be indirectly predicted with the probability of missingness.
MAR	maximum likelihood; multiple imputation; ITree method; KNN method and their variants; KMI; FCMimpute; SVD; GTD method; BP neural networks; RRP; MMF; MPT; hot/cold imputation; expectation maximization; Bayesian estimation; unconditional mean imputation; conditional mean imputation; ANN.	
MNAR	selection model; pattern mixture models; maximum likelihood; multiple imputation; ITree method; KNN method and their variants; KMI; FCMimpute; SVD; GTD method; BP neural networks; RRP; MMF; MPT; hot/cold imputation; expectation maximization; Bayesian estimation; unconditional mean imputation; conditional mean imputation; ANN.	

However, some devices do not have enough capabilities to perform complex data imputation methods. In order to solve this, Kim and Prabhakaran [80] presented a new method, named canonical correlation based k-weighted angular similarity (CkWAS), that maps the missing data with a reference pattern dataset to assign values to the missing data.

## 3. Sensor Data Fusion in Mobile Devices

Data fusion is a critical step in the integration of the data collected by multiple sensors. The main objective of the data fusion process is to increase the reliability of the decision that needs to be made using the data collected from the sensors, e.g., to increase the reliability of the identification of the ADL algorithm running in an off-the-shelf mobile device. If a single stream of data cannot eliminate uncertainty from the output, data fusion will use data from several sources with the goal of decreasing the uncertainty level of the output. Consequently, the data fusion increases the level of robustness of a system for the recognition of ADLs, reducing the effects of incorrect data captured by the sensors [81] or helping to compute solutions when the collected data are not usable for a specific task.

A mobile application implemented by Ma *et al*. [82] was tested in a Google Nexus 4 and uses the accelerometer, gyroscope, magnetometer and GPS receiver to evaluate the sensors’ accuracy, precision, maximum sampling frequency, sampling period, jitter and energy consumption in all of the sensors. The test results show that the built-in accelerometer and gyroscope sensor data have a standard deviation of approximately 0.1 to 0.8 units between the measured value and the real value, the compass sensor data deviate approximately three degrees in the normal sampling rate, and the GPS receiver data have a deviation lower than 10 meters. Thus, one of the working hypotheses of the research is the data collected by mobile sensors may be fused to work with more precision towards a common goal.

The data fusion may be performed with mobile applications, accessing the sensors data as a background process, processing the data and showing the results in a readable format or passing the results or the data to a central repository or central processing machine for further processing.

Durrant-Whyte *et al*. [83] described a decentralized data fusion system, which consists of a network of sensor nodes, each one with its own processing facility. This is a distributed system that does not require any central fusion or central communication facility, using Kalman filters to perform the data fusion. Other decentralized systems for data fusion have also been developed, improving some techniques and the sensors used [84].

The definition of the categories of the data fusion methods has already been been discussed by several authors [85,86,87]. According to these authors, the data fusion methods may be categorized as probabilistic, statistic, knowledge base theory and evidence reasoning methods. Firstly, probabilistic methods include Bayesian analysis of sensor values with Bayesian networks, state-space models, maximum likelihood methods, possibility theory, evidential reasoning and, more specifically, evidence theory, KNN and least square-based estimation methods, e.g., Kalman filtering, optimal theory, regularization and uncertainty ellipsoids. Secondly, statistic methods include the cross-covariance, covariance intersection and other robust statistics. Thirdly, knowledge base theory methods include intelligent aggregation methods, such as ANN, genetic algorithms and fuzzy logic. Finally, the evidence reasoning methods include Dempster-Shafer, evidence theory and recursive operators.

Depending on the research purpose of the data fusion, these methods have advantages and disadvantages presented in Table 5. The data fusion methods are influenced with the constraints in the previous execution of data acquisition, data processing and data imputation. The advantages and disadvantages also depend on the environmental scenarios and the choice of the correct method to apply for each research scenario.

**Table 5:** Advantages and disadvantages of the sensor data fusion methods.

Methods	Advantages	Disadvantages
Probabilistic methods	Provide methods for model estimation; allows unsupervised classification; estimate the state of variables; reduce errors in the fused location estimate; increase the amount of data without changing its structure or the algorithm; produce a fused covariance matrix that better reflects the expected location error.	Require *a priori* probabilistic knowledge of information that is not always available or realistic; classification depends on the starting point; unsuitable for large-scale systems; requires *a priori* knowledge of the uncertainties’ co-variance matrices related to the system model and its measurements.
Statistic methods	Accuracy improves from the reduction of the prediction error; high accuracy compared with other local estimators; robust with respect to unknown cross-covariance.	Complex and difficult computation is required to obtain the cross-variance; complexity and larger computational burden.
Knowledge base theory methods	Allows the inclusion of uncertainty and imprecision; easy to implement; learning ability; robust to noisy data and able to represent complex functions.	The knowledge extraction requires the intervention of human expertise (e.g., physicians), which takes time and/or may give rise to interpretation bias; difficulty in determining the adequate size of the hidden layer; inability to explain decisions; lack of transparency of data.
Evidence reasoning methods	Assign a degree of uncertainty to each source.	Require assigning a degree of evidence to all concepts.

In [88], data fusion methods are distributed in six categories. The categories of the data fusion methods include data in–data out, data in–feature out, feature in–feature out, feature in–decision out, decision in–decision out and data in–decision out.

Performing the data fusion process in real time can be difficult because of the large amount of data that may need to be fused. Ko *et al*. [88] proposed a framework, which used dynamic time warping (DTW), as the core recognizer to perform online temporal fusion on either the raw data or the features. DTW is a general time alignment and similarity measure for two temporal sequences. When compared to hidden Markov models (HMMs), the training and recognition procedures in DTW are potentially much simpler and faster, having a capability to perform online temporal fusion efficiently and accurately in real time.

The most used method for data fusion is the Kalman filter, developed for linear systems and then improved to a dynamically-weighted recursive least-squares algorithm [89]. However, as the sensor data are not linear, the authors in [89] used the extended Kalman filter to linearize the system dynamics and the measurement function around the expected state and then applied the Kalman filter as usual. A three-axis magnetometer and a three-axis accelerometer are used for the estimation of several movements [89].

Other systems employ variants of the Kalman filter to reduce the noise and improve the detection of movements. Zhao *et al*. [90] use the Rao-Blackwellization unscented Kalman filter (RBUKF) to fuse the sensor data of a GPS receiver, one gyroscope and one compass to improve the precision of the localization. The authors compare the RBUKF algorithm to the extended Kalman filter (EKF) and unscented Kalman filter (UKF), stating that the RBUKF algorithm improves the tracking accuracy and reduces computational complexity.

Walter *et al*. [91] created a system for car navigation by fusing sensor data on an Android smartphone, using the embedded sensors (*i.e.*, gyroscope) and data from the car (*i.e.*, speed information) to support navigation via GPS. The developed system employs a controller area network (CAN)-bus-to-Bluetooth adapter to establish a wireless connection between the smartphone and the CAN-bus of the car. The mobile application fuses the sensors’ data and implements a strap down algorithm and an error-state Kalman filter with good accuracy, according to the authors of [91].

Anther application for location inference was built by using the CASanDRA mobile OSGi (Open Services Gateway Initiative) framework using a *LocationFusion* enabler that fused the data acquired by all of the available sensors (*i.e.*, GPS, Bluetooth and WiFi) [92].

Mobile devices allow the development of context-aware applications, and these applications after use a framework for context information management. In [93], a mobile device-oriented framework for context information management to solve the problems on context shortage and communication inefficiency is proposed. The main functional components of this framework are the data collector, the context processor, the context manager and the local context consumer. The data collector acquires the data from internal or external sensors. Then, the context processor and the context manager extract and manage context. Finally, the local context consumer develops context-aware applications and provides appropriate services to the mobile users. The authors claim that this framework is able to run real-time applications with quick data access, power efficiency, personal privacy protection, data fusion of internal and external sensors and simplicity in usage.

Blum *et al*. [94] use the GPS receiver, compass and gyroscope embedded in Apple iPhone 4 (iOS) (Apple, Cupertino, CA, USA), iPhone 4s (iOS) (Apple, Cupertino, CA, USA) and Samsung Galaxy Nexus (Android) (Samsung, Seul, Korea), for the measurement of the location and augmented reality situations. Blum *et al*. analysed the position of the smartphone during the data collection, testing in three different orientation/body position combinations and in varying environmental conditions, obtaining results with location errors of 10 to 30 m (with a GPS receiver) and compass errors around 10 to 30°, with high standard deviations for both.

In [95], the problems of data fusion, placement and positioning of fixed and mobile sensors are focused on, presenting a two-tiered model. The algorithm combines the data collected by fixed sensors and mobile sensors, obtaining good results. The positioning of the sensors is very important, and the study implements various optimization models, ending with the creation of a new model for the positioning of sensors depending on their types. Other models are also studied by the authors, which consider the simultaneous deployment of different types of sensors, making use of more detailed sensor readings and allowing for dependencies among sensor readings.

Haala and Böhm [96] created a low-cost system using an off-the-shelf mobile device with several sensors embedded. The data collected by a GPS receiver, a digital compass and a 3D CAD model of a region are used for provisioning data related to urban environments, detecting the exact location of a building in a captured image and the orientation of the image.

A different application where data fusion is required is the recognition of physical activity. The most commonly-used sensors for the recognition of physical activity include the accelerometer, the gyroscope and the magnetic sensor. In [26], data acquired with these sensors are fused with a combined algorithm, composed of Fisher’s discriminant ratio criterion and the J3 criterion for feature selection [97]. The collection of data related to the physical activity performed is very relevant to analyse the lifestyle and physiological characteristics of people [98].

Some other applications analyse the user’s lifestyle by fusing the data collected by the sensors embedded in mobile devices. Yi *et al*. [99] presented a system architecture and a design flow for remote user physiological data and movement detection using wearable sensor data fusion.

In the medical scope, Stopczynski *et al*. [27] combined low-cost wireless EEG sensors with smartphone sensors, creating 3D EEG imaging, describing the activity of the brain. Glenn and Monteith [100] implemented several algorithms for the analysis of mental status, by using smartphone sensors. These algorithms fuse the data acquired from all of the sensors with other data obtained over Internet, to minimize the constraints of mobile sensors data.

The system proposed in [101] makes use of biosensors for different measurements, such as a surface plasmon resonance (SPR) biosensor, a smart implantable biosensor, a carbon nanotube (CNT)-based biosensor, textile sensors and enzyme-based biosensors, combined with smartphone-embedded sensors and applying various techniques for the fusion of the data and to filter the inputs to reduce the effects of the position of the device.

Social networks are widely used and promote context-aware applications, as data can be collected with the user’s mobile device sensors to promote the adaptation of the mobile applications to the user-specific lifestyle [102]. Context-aware applications are an important research topic; Andò [103] proposed an architecture to adapt context-aware applications to a real environment, using position, inertial and environmental sensors (e.g., temperature, humidity, gases leakage or smoke). The authors claim this system is very useful for the management of hazardous situations and also to supervise various physical activities, fusing the data in a specific architecture.

Other authors [104] have been studying this topic with mobile web browsers, using the accelerometer data and the positional data, implementing techniques to identify different ADLs. These systems capture the data while the user is accessing the Internet from a mobile device, analysing the movements and the distance travelled during that time and classifying the ADLs performed.

Steed and Julier [105] created the concept of *behaviour–aware sensor fusion* that uses the redirected pointing technique and the yaw fix technique to increase the usability and speed of interaction in exemplar mixed-reality interaction tasks.

In [106], vision sensors (e.g., a camera) are used and combined with accelerometer data to apply the depth from focus (DFF) method, which was limited to high precision camera systems for the detection of movements in augmented reality systems. The vision systems are relevant, as the obtained information can identify more accurately a moving object. Some mobile devices integrate RFID readers or cameras to fetch related information about objects and initiate further actions.

Rahman *et al*. [107] use a spatial-geometric approach for interacting with indoor physical objects and artefacts instead of RFID-based solutions. It uses a fusion between the data captured by an infrared (IR) camera and accelerometer data, where the IR cameras are used to calculate the 3D position of the mobile phone users, and the accelerometer in the phone provides its tilting and orientation information. For the detection of movement, they used geometrical methods, improving the detection of objects in a defined space.

Grunerbl *et al*. [108] report fusing data from acoustic, accelerometer and GPS sensors. The extraction of features from acoustic sensors uses low-level descriptors, such as root-mean-square (RMS) frame energy, mel-frequency cepstral coefficients (MFCC), pitch frequency, harmonic-to-noise ratio (HNR) and zero-crossing-rate (ZCR). They apply the naive Bayes classifier and other pattern recognition methods and report good results in several situations of daily life.

Gil *et al*. [109] present other systems to perform sensor fusion, such as LifeMap, which is a smartphone-based context provider for location-based services, as well as the Joint Directors of Laboratories (JDL) model and waterfall IF model, which define various levels of abstraction for the sensor fusion techniques. Gil *et al*. also present the *inContexto* system, which makes use of embedded sensors, such as the accelerometer, digital compass, gyroscope, GPS, microphone and camera, applying several filters and techniques to recognize physical actions performed by users, such as walking, running, standing and sitting, and also to retrieve context information from the user.

In [110], a sensor fusion-based wireless walking-in-place (WIP) interaction technique is presented, creating a human walking detection algorithm based on fusing data from both the acceleration and magnetic sensors integrated in a smartphone. The proposed algorithm handles a possible data loss and random delay in the wireless communication environment, resulting in reduced wireless communication load and computation overhead. The algorithm was implemented for mobile devices equipped with magnetic, accelerometer and rotation (gyroscope) sensors. During the tests, the smartphone was adapted to the user’s leg. After some tests, the authors decided to implement the algorithm with two smartphones and a magnetometer positioned on the user’s body, combining the magnetic sensor-based walking-in-place and acceleration-based walking in place, in order to discard the use of a specific model (e.g., gait model) and a historical data accumulated. However, the acceleration-based technique does not support the correct use in slow-speed walking, and the magnetic sensor-based technique does not support both normal and fast walking speeds.

Related to the analysis of walking, activities and movements, several studies have employed the GPS receiver, combined with other sensors, such as the accelerometry, magnetometer, rotation sensors and others, with good accuracy using some fusion algorithms, such as the naive and oracle methods [111], machine learning methods and kinematic models [112] and other models adapted to mobile devices [91]. The framework implemented by Tsai *et al*. [113] detects the physical activity fusing data from several sensors embedded in the mobile device and using crowdsourcing, based on the methods’ result of the merging of classical statistical detection and estimation theory and uses value fusion and decision fusion of human sensor data and physical sensor data.

In [114], Lee and Chung created a system with a data fusion approach based on several discrete data types (e.g., eye features, bio-signal variation, in-vehicle temperature or vehicle speed) implemented in an Android smartphone, allowing high resolution and flexibility. The study involves different sensors, including video, ECG, photoplethysmography, temperature and a three-axis accelerometer, which are assigned as input sources to an inference analysis framework. A fuzzy Bayesian network includes the eyes feature, bio-signal extraction and the feature measurement method.

In [81], a sensor weighted network classifier (SWNC) model is proposed, which is composed of three classification levels. A set of binary activity classifiers consists of the first classification level of the model proposed. The second classification level is defined by node classifiers, which are decision making models. The decisions of the model are combined through a class-dependent weighting fusion scheme structure with a structure defined through several base classifiers. The weighted decisions obtained by each node classifier are fused on the model proposed. Finally, the last classification level has a similar constitution of the second classification level. Independently of the level of noise imposed, when the mobile device stays in a static position during the data acquisition process, a performance up to 60% can be achieved with the proposed method.

Chen *et al*. [115] and Sashima *et al*. [116] created client-server platforms that monitor the activities in a space with a constant connection with a server. This causes major energy consumption and decreases the capabilities of the mobile devices in the detection of the activities performed.

Another important measurement with smart phone sensors is related to the orientation of the mobile device. Ayub *et al*. [117] have already implemented the DNRF (drift and noise removal filter) with a sensor fusion of gyroscope, magnetometer and accelerometer data to minimize the drift and noise in the output orientation.

Other systems have been implemented for gesture recognition. Zhu *et al*. [118] proposed a high-accuracy human gesture recognition system based on multiple motion sensor fusion. The method reduces the energy overhead resulting from frequent sensor sampling and data processing with a high energy-efficient very large-scale integration (VLSI) architecture. The results obtained have an average accuracy for 10 gestures of 93.98% for the user-independent case and 96.14% for the user-dependent case.

As presented in Section 2.4, decentralized systems, cloud-based systems or server-side systems are used for data processing. As the data fusion is the next stage, van de Ven *et al*. [119] presented the Complete ambient assisting living experiment (CAALYX) system that provides continuous monitoring of people’s health. It has software installed on the mobile phone that uses data fusion for decision support to trigger additional measurements, classify health conditions or schedule future observations.

In [120], Chen *et al*. presented a decentralized data fusion and active sensing (D^2^FAS) algorithm for mobile sensors to actively explore the road network to gather and assimilate the most informative data for predicting the traffic phenomenon.

Zhao *et al*. [121,122] developed the *COUPON* (Cooperative Framework for Building Sensing Maps in Mobile Opportunistic Networks) framework, a novel cooperative sensing and data forwarding framework to build sensing maps satisfying specific sensing quality with low delay and energy consumption. This framework implements two cooperative forwarding schemes by leveraging data fusion; these are epidemic routing with fusion (ERF) and binary spray-and-wait with fusion (BSWF). It considers that packets are spatial-temporally correlated in the forwarding process and derives the dissemination law of correlated packets. The work demonstrates that the cooperative sensing scheme can reduce the number of samplings by 93% compared to the non-cooperative scheme; ERF can reduce the transmission overhead by 78% compared to epidemic routing (ER); BSWF can increase the delivery ratio by 16% and reduce the delivery delay and transmission overhead by 5% and 32%, respectively, compared to binary spray-and-wait (BSW).

In [123], a new type of sensor node is described with modular and reconfigurable characteristics, composed of a main board, with a processor, FR (Frequency Response) circuits and a power supply, as well as an expansion board. A software component was created to join all sensor data, proposing an adaptive processing mechanism.

In [124], the C-SPINE (Collaborative-Signal Processing in Node Environment) framework is proposed, which uses multi-sensor data fusion among CBSNs (Collaborative Body Sensor Networks) to enable joint data analysis, such as filtering, time-dependent data integration and classification, based on a multi-sensor data fusion schema to perform automatic detection of handshakes between two individuals and to capture possible heart rate-based emotional reactions due to individuals meeting.

A wireless, wearable, multi-sensor system for locomotion mode recognition is described in [125], with three inertial measurement units and eight force sensors, measuring both kinematic and dynamic signals of the human gait. The system uses a linear discriminant analysis classifier, obtaining good results for motion mode recognition during the stance phase, during the swing phase and for sit-to-stand transition recognition.

Chen [126] developed an algorithm for data fusion to track both non-manoeuvring and manoeuvring targets with mobile sensors deployed in a wireless sensor network (WSN). It applies the *GATING* technique to solve the problem of mobile-sensor data fusion tracking (MSDF) for targets. In WSNs, an adaptive filter (Kalman filter) is also used, consisting of a data association technique denoted as one-step conditional maximum likelihood.

Other studies have focused on sensor fusion for indoor navigation [127,128]. Saeedi *et al*. [127] proposes a context-aware personal navigation system (PNS) for outdoor personal navigation using a smartphone. It uses low-cost sensors in a multi-level fusion scheme to improve the accuracy and robustness of the context-aware navigation system [127]. The system developed has several challenges, such as context acquisition, context understanding and context-aware application adaptation, and it is mainly used for the recognition of the people’s activities [127]. It uses the accelerometer, gyroscope and magnetometer sensors and the GPS receiver available on the off-the-shelf mobile devices to detect and recognize the motion of the mobile device, the orientation of the mobile device and the location and context of the data acquisition [127]. The system includes a feature-level fusion scheme to recognize context information, which is applied after the data are processed and the signal’s features are extracted [127]. Bhuiyan *et al*. [128] evaluate the performance of several methods for multi-sensor data fusion focused on a Bayesian framework. A Bayesian framework consists of two steps [128], such as prediction and correction. In the prediction stage, the current state is updated based on the previous state and the system dynamics [128]. In the correction stage, the prediction is updated with the new measurements [128]. In [128], the authors studied different combinations of methods for data fusion, such as a linear system and Kalman filter and a non-linear system and extended Kalman filter, implementing some sensor data fusion systems with good results.

In [129], a light, high-level fusion algorithm to detect the daily activities that an individual performs is presented. The proposed algorithm is designed to allow the implementation of a context-aware application installed on a mobile device, working with minimum computational cost. The quality of the estimation of the ADLs depends on the presence of biometric information and the position and number of available inertial sensors. The best estimation for continuous physical activities obtained, with the proposed algorithm, is approximately 90%.

The *CHRONIOUS* system is developed for smart devices as a decision support system, integrating a classification system with two parallel classifiers. It combines an expert system (rule-based system) and a supervised classifier, such as SVM, random forests, ANN (e.g., the multi-layer perceptron), decision trees and naive Bayes [130]. Other systems for the recognition of ADLs have also been implemented using other classifiers during the data fusion process, e.g., decision tables.

Martín *et al*. [131] evaluated the accuracy, computational costs and memory fingerprints in the classifiers mentioned above working with different sensor data and different optimization. The system that implements these classifiers encompasses different sensors, such as acceleration, gravity, linear acceleration, magnetometer and gyroscope, with good results, as presented in [131]. Other systems were implemented using lightweight methods for sensor data fusion, e.g., the KNOWME system [132], which implements the autoregressive-correlated Gaussian model for data classification and data fusion.

Other research studies focused on the compensation of the ego-motion of the camera, carried out with the data fusion of Viola and Jones face detector and inertial sensors, reporting good results [133].

In [134], sensor fusion is used for error detection, implementing the fusion of ECG with the blood pressure signal, blood pressure with body temperature and acceleration data with ECG.

In [135], Jin *et al*. proposed a robust dead-reckoning (DR) pedestrian tracking system to be used with off-the-shelf sensors. It implements a robust tracking task as a generalized maximum *a posteriori* sensor fusion problem, and then, they narrow it to a simple computation procedure with certain assumptions, with a reduction in average tracking error up to 73.7%, compared to traditional DR tracking methods.

**Table 6:** Examples of sensor data fusion methods.

Sensors	Methods	Achievements
Accelerometer; Gyroscope; Magnetometer; Compass; GPS receiver; Bluetooth; Wi-Fi; digital camera; microphone; RFID readers; IR camera.	DR pedestrian tracking system; Autoregressive-Correlated Gaussian Model; CASanDRA mobile OSGi framework; Genetic Algorithms; Fuzzy Logic; Dempster-Shafer; Evidence Theory; Recursive Operators; DTW framework; *CHRONIOUS* system; SVM; Random Forests; ANN; Decision Trees; Naive Bayes; Decision Tables; Bayesian analysis.	The use of several sensors reduces the noise effects; these methods also evaluated the accuracy of sensor data fusion; the data fusion may be performed with mobile applications, accessing the sensors data as a background process, processing the data and showing the results in a readable format.
Accelerometer; Gyroscope; Magnetometer; Compass; GPS receiver; Bluetooth; WiFi; digital camera; microphone; low-cost wireless EEG sensors; RFID readers; IR camera	Kalman Filtering; C-SPINE framework; DNRF method; SWNC model; *GATING* technique; *COUPON* framework; CAALYX system; high energy-efficient very large-scale integration (VLSI) architecture; sensor-fusion-based wireless walking-in-place (WIP) interaction technique; J3 criterion; DFF method; ERF method; BSWF method; *inContexto* system; RMS frame energy; MFCC method; pitch frequency; HNR method; ZCR method; KNN; Least squares-based estimation methods; Optimal Theory, Regularization; Uncertainty Ellipsoids.	These methods allow a complex processing of amount of data acquired, because it is central processed in a server-side system; using data from several sources decreases the uncertainty level of the output; performing the data fusion process in real time can be difficult because of the large amount of data that may need to be fused; the data fusion may be performed with mobile applications, accessing the sensors data as a background process, processing the data and showing the results in a readable format or passing the results or the data to a central repository or central processing machine for further processing.
Gyroscope; Compass; Magnetometer; GPS receiver.	Kalman Filtering; Bayesian analysis.	It is mainly useful for the context-aware localization systems; defined several recognizer algorithms to perform online temporal fusion on either the raw data or the features.
ECG and others	Kalman Filtering.	Using data from several sources decreases the uncertainty level of the output; defined several recognizer algorithms to perform online temporal fusion on either the raw data or the features.

In [136], Grunerbl *et al*. developed a smartphone-based recognition of states and state changes in bipolar disorder patients, implemented an optimized state change detection, developing various fusion methods with different strategies, such as logical AND, OR, and their own weighted fusion, obtaining results with good accuracy.

In Table 6, a summary of sensor data fusion techniques, their achievements and sensors used is presented. This table also helps make clear that different methods can be applied to different types of data collected by different sensors.

In conclusion, sensor fusion techniques for mobile devices are similar to those employed with other external sensors, because the applications usually involve embedded and external sensors at the same time. The Kalman filter is the most commonly used, but on these devices, there is a need to use low processing techniques. Some research applications need a high processing capacity, and in this case, the mobile device is only used to capture the data. After capturing the data, the captured data will be sent to a server for later processing. The most important criterion for choosing a data fusion method should be based on the limited capacities of the mobile devices, as these devices increase the possibility to collect physiological data anywhere and at anytime with low cost.

## 4. Application in Ambient Assisted Living

As described in this paper, the use of data fusion techniques with sensors available in off-the-shelf mobile devices may be shaped to form different types of systems and architectures. These techniques are implementable in systems based on mobile technologies, with local processing, server-side processing or a mix of these, or as distributed systems that work as a cooperative system collecting the data with different mobile devices.

Mobile systems for the recognition of ADLs have two possible architectures, are where all processing activities are performed locally on the mobile device or another where the data processing will be executed totally, or in part, on a remote machine. Several authors have developed algorithms related to context-aware sensing (e.g., environment recognition, gestural recognition and browsing history) [88,93,104,109,115], positioning of objects [107] and localization sensing [82,90,91,94,137].

Several works have focused on the development of different methods to recognize some ADLs [92,111,112,129,132,138,139], such as sitting, walking, running, cycling, standing still, climbing stairs, using the lift, getting up, falling and having a meeting. In addition, off-the-shelf mobile devices have several sensors embedded that are capable of recognizing psychological/emotional states, including the recognition of mental stress [140].

The recognition of the ADLs [81,110,131,141,142] is very important in systems developed to support and monitor elderly people or people with some disease or impairment [143,144]. When combined with the user’s agenda, they can be employed for monitoring and learning lifestyles and physical exercise, helping people in emergency situations [116,136] and other situations where the sensors can improve life quality, e.g., driving monitoring [114].

Healthcare is one of the most important purposes of the recognition of the ADLs, and commonly, it is performed in smart health environments [4,145], with several technologies that include the relation between several mobile devices capturing the sensors’ data connected by WSNs and using sensor webs to aggregate the data collected by several sensors and identify the ADLs with more accuracy [146].

Distributed systems for sensor data fusion with off-the-shelf mobile devices have been used for tracking [120,121,122,126,147], location detection [83,84,123,124,148,149], health monitoring [99,103,119], user monitoring [113,150], automotive systems [151] and other purposes.

In [152], several types of sensors (e.g., complementing the detection of objects using a camera and inertial sensors) are used to classify objects and detect changes. Using mobile devices to capture sensors’ signals, a problem with the involuntary movements of the mobile device during the performance of some ADLs is very high. However, the use of data fusion technologies allows one to minimize the effects of the possible involuntary movements of the mobile device, commonly considered noise [117].

In conclusion, the sensor fusion techniques are very useful to improve the recognition of the ADLs, which is the main goal of this review. Moreover, the identification of a wide range of ADLs by a mobile device is a major milestone on the development of a personal digital life coach [153]. The sensor fusion techniques increase the reliability of these systems, allowing, e.g., to learn the different peoples’ lifestyles. Many mobile applications send data to a remote computer that carries out the processing and sends back the results to the mobile device. However, the local processing is faster than server-side processing, reducing the bandwidth usage during all processes.

## 5. Conclusions

Data acquisition, data processing, data imputation on one sensor data stream and, finally, multiple sensor data fusion together are the proposed roadmap to achieve a concrete task, and as discussed in this paper, the task at hand is the identification of activities of daily living. While these steps pose little challenge when performed in fixed computational environments, where resources are virtually illimited, when their execution is intended in mobile off-the-shelf devices, a new type of challenges arises with the restrictions of the computational environment.

The use of mobile sensors requires a set of techniques to classify and to process the data acquired to make them usable by software components and to automate the execution of specific tasks. The data pre-processing and data cleaning tasks are performed at the start of the sensors’ data characterization. The collected data may have inconsistent and/or unusable data, normally called environmental noise. However, the application of filters helps with removing the unusable data. The most commonly-used filters are the Kalman filter and its variants, as these methods are often reported to have good accuracy. Nevertheless, it must be noted that the correct definition of the important features of the collected data influences the correct measurement of the accuracy of the Kalman filter. Therefore, and because it is difficult to assess the accuracy of one particular type of Kalman filter, especially when used regarding the identification of activities of daily living, it is advisable to explore different sensor data fusion technologies. The main methods for the identification of the different features of the sensor signal are machine learning and pattern recognition techniques.

Sensor fusion methods are normally classified into four large groups: probabilistic methods, statistical methods, knowledge-based theory methods and evidence reasoning methods. Although several methods have been studied and discussed in this paper, the choice of the best method for each purpose depends on the quantity and types of sensors used, on the diversity in the representation of the data, on the calibration of the sensors, on the limited interoperability of the sensors, on the constraints in the statistical models and, finally, on the limitations of the implemented algorithms.

Sensors available in off-the-shelf mobile devices can support the implementation of sensor fusion techniques and improve the reliability of the algorithms created for these devices. However, mobile devices have limited processing capacity, memory and autonomy. Nevertheless, the fusion of the sensors’ data may be performed with mobile applications, which access the sensors’ data as a background task, processing the data collected and showing the results in a readable format to the user or sending the results or the data to a central repository or central processing machine.

The techniques related to the concepts of sensor and multi-sensor data fusion presented in this paper have different purposes, including the detection/identification of activities of daily living and other medical applications. For off-the-shelf mobile devices, the positioning of the device during the data acquisition process is in itself an additional challenge, as the accuracy, precision and usability of obtained data are also a function of the sensors’ location. Therefore, it has to be expected that these systems can fail only because of poor mobile device positioning.

Several research studies have been carried out regarding sensor fusion techniques applied to the sensors available in off-the-shelf mobile devices, but most of them only detect basic activities, such as walking. Due to the large market of mobile devices, e.g., smartphones, tablets or smartwatches, ambient assisted living applications on these platforms become relevant for a variety of purposes, including tele-medicine, monitoring of elderly people, monitoring of sport performance and other medical, recreational, fitness or leisure activities. Moreover, the identification of a wide range of activities of daily living is a milestone in the process of building a personal digital life coach.

The applicability of sensor data fusion techniques for mobile platforms is therefore dependent on the variable characteristics of the mobile platform itself, as these are very diverse in nature and features, from local storage capability to local processing power, battery life or types of communication protocols. Nevertheless, experimenting with the algorithms and techniques described previously, so as to adapt them to a set of usage scenarios and a class of mobile devices, will render these techniques usable in most mobile platforms without major impairments.

## References

[1] Yin G., Bruckner D. (2010). Daily activity learning from motion detector data for Ambient Assisted Living. Technological Innovation for Sustainability.

[2] Memon M., Wagner S.R., Pedersen C.F., Beevi F.H., Hansen F.O. (2014). Ambient assisted living healthcare frameworks, platforms, standards, and quality attributes. Sensors.

[3] Siegel C., Hochgatterer A., Dorner T.E. (2014). Contributions of ambient assisted living for health and quality of life in the elderly and care services–A qualitative analysis from the experts’ perspective of care service professionals. BMC Geriatr..

[4] Holzinger A., Röcker C., Ziefle M. (2015). From Smart Health to Smart Hospitals. Smart Health: State-of-the-Art and Beyond, Springer Lecture Notes in Computer Science, LNCS 8700.

[5] Developers A. Sensors Overview | Android Developers 2015. http://developer.android.com/guide/topics/sensors/sensors_overview.html.

[6] Apple Apple - iPhone 6 - Technical Specifications 2015. http://www.apple.com/iphone-6/specs/.

[7] Santochi M., Dini G. (1998). Sensor Technology in Assembly Systems. CIRP Ann. Manuf. Technol..

[8] White R. (1987). A Sensor Classification Scheme. IEEE Trans. Ultrason. Ferroelectr. Freq. Control.

[9] Roebuck A., Monasterio V., Gederi E., Osipov M., Behar J., Malhotra A., Penzel T., Clifford G.D. (2014). A review of signals used in sleep analysis. Physiol. Meas..

[10] Preece S.J., Goulermas J.Y., Kenney L.P., Howard D., Meijer K., Crompton R. (2009). Activity identification using body mounted sensors: a review of classification techniques. Physiol. Meas..

[11] Mumolo E., Nolich M., Vercelli G. (2003). Algorithms for acoustic localization based on microphone array in service robotics. Robot. Auton. Syst..

[12] Guan D., Yuan W., Jehad Sarkar A.M., Ma T., Lee Y.K. (2011). Review of Sensor-based Activity Recognition Systems. IETE Techn. Rev..

[13] Gentili M., Mirchandani P.B. (2012). Locating sensors on traffic networks: Models, challenges and research opportunities. Transp. Res. Part C: Emerg. Technol..

[14] Lee S., Ozsecen M., Della Toffola L., Daneault J.F., Puiatti A., Patel S., Bonato P. Activity detection in uncontrolled free-living conditions using a single accelerometer. Proceedings of the 2015 IEEE 12th International Conference on Wearable and Implantable Body Sensor Networks (BSN).

[15] Scheeper P.R., van der Donk A.G.H., Olthuis W., Bergveld P. (1994). A review of silicon microphones. Sens. Actuators A Phys..

[16] Hoummady M., Campitelli A., Wlodarski W. (1997). Acoustic wave sensors: Design, sensing mechanisms and applications. Smart Mater. Struct..

[17] Wilson A.D., Baietto M. (2009). Applications and advances in electronic nose technologies. Sensors.

[18] Suzuki T., Nakauchi Y. Intelligent medicine case that monitors correct dosing. Proceedings of the 2010 7th International Symposium on Mechatronics and Its Applications (ISMA).

[19] Hill E.W., Vijayaragahvan A., Novoselov K. (2011). Graphene Sensors. IEEE Sens. J..

[20] Lenz J., Edelstein S. (2006). Magnetic sensors and their applications. IEEE Sens. J..

[21] Allen J. (2007). Photoplethysmography and its application in clinical physiological measurement. Physiol. Meas..

[22] Deng K.-L. Next generation fiber optic sensing and its applications. Proceedings of the International Conference on Wireless and Optical Communications.

[23] Khusainov R., Azzi D., Achumba I.E., Bersch S.D. (2013). Real-time human ambulation, activity, and physiological monitoring: taxonomy of issues, techniques, applications, challenges and limitations. Sensors.

[24] Zhang L., Liu J., Jiang H., Guan Y. (2013). SensTrack: Energy-Efficient Location Tracking With Smartphone Sensors. IEEE Sens. J..

[25] Novak D., Rebersek P., De Rossi S.M., Donati M., Podobnik J., Beravs T., Lenzi T., Vitiello N., Carrozza M.C., Munih M. (2013). Automated detection of gait initiation and termination using wearable sensors. Med. Eng. Phys..

[26] He Y., Li Y. (2013). Physical Activity Recognition Utilizing the Built-In Kinematic Sensors of a Smartphone. Int. J. Distrib. Sens. Netw..

[27] Stopczynski A., Stahlhut C., Larsen J.E., Petersen M.K., Hansen L.K. (2014). The smartphone brain scanner: A portable real time neuroimaging system. PLoS ONE.

[28] Daponte P., de Vito L., Picariello F., Riccio M. (2013). State of the art and future developments of measurement applications on smartphones. Measurement.

[29] Scalvini S., Baratti D., Assoni G., Zanardini M., Comini L., Bernocchi P. (2013). Information and communication technology in chronic diseases: A patient’s opportunity. J. Med. Person.

[30] Bersch S.D., Azzi D., Khusainov R., Achumba I.E., Ries J. (2014). Sensor data acquisition and processing parameters for human activity classification. Sensors.

[31] Lim L., Misra A., Mo T. (2012). Adaptive data acquisition strategies for energy-efficient, smartphone-based, continuous processing of sensor streams. Distrib. Parallel Databases.

[32] Paucher R., Turk M. Location-based augmented reality on mobile phones. Proceedings of the 2010 IEEE Computer Society Conference on Computer Vision and Pattern Recognition Workshops (CVPRW).

[33] Misra A., Lim L. Optimizing Sensor Data Acquisition for Energy-Efficient Smartphone-Based Continuous Event Processing. Proceedings of the 2011 12th IEEE International Conference on Mobile Data Management (MDM).

[34] Kang S., Lee Y., Min C., Ju Y., Park T., Lee J., Rhee Y., Song J. Orchestrator: An active resource orchestration framework for mobile context monitoring in sensor-rich mobile environments. Proceedings of the 2010 IEEE International Conference on Pervasive Computing and Communications (PerCom).

[35] Agrawal S., Kamal R. (2015). Computational Orchestrator: A Super Class for Matrix, Robotics and Control System Orchestration. Int. J. Comput. Appl..

[36] Vallina-Rodriguez N., Crowcroft J. ErdOS: Achieving Energy Savings in Mobile OS. Proceedings of the Sixth International Workshop on MobiArch (MobiArch’11).

[37] Priyantha B., Lymberopoulos D., Liu J. (2011). LittleRock: Enabling Energy-Efficient Continuous Sensing on Mobile Phones. IEEE Pervasive Comput..

[38] Lu H., Yang J., Liu Z., Lane N.D., Choudhury T., Campbell A.T. The Jigsaw Continuous Sensing Engine for Mobile Phone Applications. Proceedings of the 8th ACM Conference on Embedded Networked Sensor Systems (SenSys’10).

[39] Rachuri K.K., Mascolo C., Musolesi M., Rentfrow P.J. SociableSense: Exploring the Trade-offs of Adaptive Sampling and Computation Offloading for Social Sensing. Proceedings of the 17th Annual International Conference on Mobile Computing and Networking (MobiCom’11).

[40] Deshpande A., Guestrin C., Madden S.R., Hellerstein J.M., Hong W. Model-driven Data Acquisition in Sensor Networks. Proceedings of the Thirtieth International Conference on Very Large Data Bases-Volume 30. VLDB Endowment (VLDB’04).

[41] Reilent E., Loobas I., Pahtma R., Kuusik A. Medical and context data acquisition system for patient home monitoring. Proceedings of the 2010 12th Biennial Baltic Electronics Conference (BEC).

[42] Marzencki M., Hung B., Lin P., Huang Y., Cho T., Chuo Y., Kaminska B. Context-aware physiological data acquisition and processing with wireless sensor networks. Proceedings of the 2010 IEEE International Workshop on Medical Measurements and Applications Proceedings (MeMeA).

[43] Agoston K., Nagy C. Data acquisition for angular measuring and positioning system. Proceedings of the 2012 IEEE International Conference on Automation Quality and Testing Robotics (AQTR).

[44] Harvey P., Woodward B., Datta S., Mulvaney D. (2011). Data acquisition in a wireless diabetic and cardiac monitoring system. IEEE Eng. Med. Biol. Soc. Conf. Proc..

[45] Bieber G., Haescher M., Vahl M. Sensor requirements for activity recognition on smart watches. Proceedings of the 6th International Conference on PErvasive Technologies Related to Assistive Environments.

[46] Sorber J.M., Shin M., Peterson R., Kotz D. Plug-n-trust: Practical trusted sensing for mhealth. Proceedings of the 10th International Conference on Mobile Systems, Applications, and Services.

[47] Zhu W., Liu L., Yin S., Hu S., Tang E.Y., Wei S. (2013). Motion-sensor fusion-based gesture recognition and its VLSI architecture design for mobile devices. Int. J. Electron..

[48] Lane N., Miluzzo E., Lu H., Peebles D., Choudhury T., Campbell A. (2010). A survey of mobile phone sensing. IEEE Commun. Mag..

[49] Kim T.H., Adeli H., Robles R.J., Balitanas M. (2011). Ubiquitous Computing and Multimedia Applications.

[50] Hwang Y.C., Oh R.D., Ji G.H. (2011). A Sensor Data Processing System for Mobile Application Based Wetland Environment Context-aware. Ubiquitous Computing and Multimedia Applications.

[51] Choi M. (2012). A Platform-Independent Smartphone Application Development Framework. Computer Science and Convergence.

[52] Bersch S.D., Azzi D., Khusainov R., Achumba I.E., Ries J. (2014). Sensor data acquisition and processing parameters for human activity classification. Sensors.

[53] Pejovic V., Musolesi M. (2015). Anticipatory Mobile Computing. ACM Comput. Surv..

[54] Imai S., Miyamoto M., Arai Y., Inomata T. A data processing method for motion estimation considering network and sensor node loads. Proceedings of the 2012 IEEE 11th International Conference on Cognitive Informatics & Cognitive Computing (ICCI*CC).

[55] Pombo N., Garcia N., Felizardo V., Bousson K. Big data reduction using RBFNN: A predictive model for ECG waveform for eHealth platform integration. Proceedings of the 2014 IEEE 16th International Conference on e-Health Networking, Applications and Services (Healthcom).

[56] Yamada S., Watanabe Y., Kitagawa H., Amagasa T. Location-Based Information Delivery Using Stream Processing Engine. Proceedings of the 7th International Conference on Mobile Data Management (2006 MDM).

[57] Lin F.X., Rahmati A., Zhong L. Dandelion: A framework for transparently programming phone-centered wireless body sensor applications for health. Proceedings of the WH’10 Wireless Health 2010.

[58] Dolui K., Mukherjee S., Datta S.K. Smart Device Sensing Architectures and Applications. Proceedings of the 2013 International Computer Science and Engineering Conference (Icsec).

[59] Imai S., Miyamoto M., Arai Y., Inomata T. Sensor Data Processing Method Based on Observed Person’s Similarity for Motion Estimation. Proceedings of the 2013 IEEE 27th International Conference on Advanced Information Networking and Applications Workshops (Waina).

[60] Gurrin C., Qiu Z., Hughes M., Caprani N., Doherty A.R., Hodges S.E., Smeaton A.F. (2013). The smartphone as a platform for wearable cameras in health research. Am. J. Prev. Med..

[61] Postolache O., Girao P.S., Ribeiro M., Guerra M., Pincho J., Santiago F., Pena A. Enabling telecare assessment with pervasive sensing and Android OS smartphone. Proceedings of the 2011 IEEE International Workshop on Medical Measurements and Applications Proceedings (MeMeA).

[62] Wang G., Zimmermann R. Spatial sensor data processing and analysis for mobile media applications. Proceedings of the 1st ACM SIGSPATIAL PhD Workshop.

[63] Vateekul P., Sarinnapakorn K. Tree-Based Approach to Missing Data Imputation. Proceedings of the IEEE International Conference on Data Mining Workshops (ICDMW’09).

[64] D’Ambrosio A., Aria M., Siciliano R. (2012). Accurate Tree-based Missing Data Imputation and Data Fusion within the Statistical Learning Paradigm. J. Classif..

[65] Huang X.Y., Li W., Chen K., Xiang X.H., Pan R., Li L., Cai W.X. (2013). Multi-matrices factorization with application to missing sensor data imputation. Sensors.

[66] García-Laencina P.J., Sancho-Gómez J.L., Figueiras-Vidal A.R., Verleysen M. (2009). K nearest neighbours with mutual information for simultaneous classification and missing data imputation. Neurocomputing.

[67] Ni D., Leonard J.D., Guin A., Feng C. (2005). Multiple Imputation Scheme for Overcoming the Missing Values and Variability Issues in ITS Data. J. Transp. Eng..

[68] Smith B., Scherer W., Conklin J. (2003). Exploring Imputation Techniques for Missing Data in Transportation Management Systems. Transp. Res. Rec..

[69] Qu L., Zhang Y., Hu J., Jia L., Li L. A BPCA based missing value imputing method for traffic flow volume data. Proceedings of the 2008 IEEE Intelligent Vehicles Symposium.

[70] Jiang N., Gruenwald L. Estimating Missing Data in Data Streams. Proceedings of the 12th International Conference on Database Systems for Advanced Applications (DASFAA 2007).

[71] Ling W., Dong-Mei F. Estimation of Missing Values Using a Weighted K-Nearest Neighbors Algorithm. Proceedings of the International Conference on Environmental Science and Information Application Technology (ESIAT 2009).

[72] Hruschka E.R., Hruschka E.R., Ebecken N.F.F. (2004). Towards Efficient Imputation by Nearest-Neighbors: A Clustering-Based Approach. AI 2004: Advances in Artificial Intelligence.

[73] Luo J., Yang T., Wang Y. Missing value estimation for microarray data based on fuzzy C-means clustering. Proceedings of the Eighth International Conference on High-Performance Computing in Asia-Pacific Region.

[74] Smaragdis P., Raj B., Shashanka M. (2010). Missing Data Imputation for Time-Frequency Representations of Audio Signals. J. Signal Process. Syst..

[75] Liu Y., Lv Z., Wang W. (2013). An Improved Generalized-Trend-Diffusion-Based Data Imputation for Steel Industry. Math. Probl. Eng..

[76] Iacus S.M., Porro G. (2007). Missing data imputation, matching and other applications of random recursive partitioning. Comput. Stat. Data Anal..

[77] Bruni R. (2004). Discrete models for data imputation. Discret. Appl. Math..

[78] Pombo N.G.C.C. (2014). Information Technologies for Pain Management. Ph.D. Thesis.

[79] Krichmar J.L., Snook J.A. (2002). A neural approach to adaptive behavior and multi-sensor action selection in a mobile device. Rob. Autom..

[80] Kim D.J., Prabhakaran B. Faulty and Missing Body Sensor Data Analysis. Proceedings of the 2013 IEEE International Conference on Healthcare Informatics (ICHI).

[81] Banos O., Damas M., Pomares H., Rojas I. (2012). On the use of sensor fusion to reduce the impact of rotational and additive noise in human activity recognition. Sensors.

[82] Ma Z., Qiao Y., Lee B., Fallon E. Experimental evaluation of mobile phone sensors. Proceedings of the Signals and Systems Conference (ISSC 2013), 24th IET Irish.

[83] Durrant-Whyte H., Stevens M., Nettleton E. Data fusion in decentralised sensing networks. Proceedings of the 4th International Conference on Information Fusion.

[84] Aziz A.M. (2014). A new adaptive decentralized soft decision combining rule for distributed sensor systems with data fusion. Inf. Sci..

[85] Akhoundi M.A.A., Valavi E. (2010). Multi-Sensor Fuzzy Data Fusion Using Sensors with Different Characteristics. ArXiv E-Prints.

[86] Khaleghi B., Khamis A., Karray F.O., Razavi S.N. (2013). Multisensor data fusion: A review of the state-of-the-art. Inf. Fusion.

[87] Pombo N., Bousson K., Araújo P., Viana J. (2015). Medical decision-making inspired from aerospace multisensor data fusion concepts. Inf. Health Soc. Care.

[88] Ko M.H., West G., Venkatesh S., Kumar M. (2008). Using dynamic time warping for online temporal fusion in multisensor systems. Inf. Fusion.

[89] Tanveer F., Waheed O.T., ur Rehman A. (2011). Design and Development of a Sensor Fusion based Low Cost Attitude Estimator. J. Space Technol..

[90] Zhao L., Wu P., Cao H. (2013). RBUKF Sensor Data Fusion for Localization of Unmanned Mobile Platform. Res. J. Appl. Sci. Eng. Technol..

[91] Walter O., Schmalenstroeer J., Engler A., Haeb-Umbach R. Smartphone-based sensor fusion for improved vehicular navigation. Proceedings of the 10th Workshop on Positioning Navigation and Communication (WPNC).

[92] Iglesias J., Cano J., Bernardos A.M., Casar J.R. A ubiquitous activity-monitor to prevent sedentariness. Proceedings of the IEEE International Conference on Pervasive Computing and Communications Workshops (PERCOM Workshops).

[93] Yongkai Z., Shuangquan W., Zhuang Z., Canfeng C., Jian M. A mobile device oriented framework for context information management. Proceedings of the IEEE Youth Conference on Information, Computing and Telecommunication.

[94] Blum J.R., Greencorn D.G., Cooperstock J.R. (2013). Smartphone Sensor Reliability for Augmented Reality Applications. Mobile and Ubiquitous Systems: Computing, Networking, and Services.

[95] Neidhardt A., Luss H., Krishnan K.R. Data fusion and optimal placement of fixed and mobile sensors. Proceedings of the IEEE Sensors Applications Symposium.

[96] Haala N., Böhm J. (2003). A multi-sensor system for positioning in urban environments. ISPRS J. Photogramm. Remote Sens..

[97] Theodoridis S. (2015). Machine Learning: A Bayesian and Optimization Perspective.

[98] Miller G. (2012). The Smartphone Psychology Manifesto. Perspect. Psychol. Sci..

[99] Yi W.J., Sarkar O., Mathavan S., Saniie J. Wearable sensor data fusion for remote health assessment and fall detection. Proceedings of the IEEE International Conference on Electro/Information Technology (EIT).

[100] Glenn T., Monteith S. (2014). New measures of mental state and behavior based on data collected from sensors, smartphones, and the Internet. Curr. Psychiatry Rep..

[101] Ponmozhi J., Frias C., Marques T., Frazão O. (2012). Smart sensors/actuators for biomedical applications: Review. Measurement.

[102] Beach A., Gartrell M., Xing X., Han R., Lv Q., Mishra S., Seada K. Fusing mobile, sensor, and social data to fully enable context-aware computing. Proceedings of the Eleventh Workshop on Mobile Computing Systems Applications.

[103] Andò B., Baglio S., Pistorio A. (2014). A Smart Multi-Sensor Approach to Monitoring Weak People in Indoor Environments. J. Sens. Technol..

[104] Phan T., Kalasapur S., Kunjithapatham A. Sensor fusion of physical and social data using Web SocialSense on smartphone mobile browsers. Proceedings of the IEEE 11th Consumer Communications and Networking Conference (CCNC).

[105] Steed A., Julier S. Behaviour-aware sensor fusion: Continuously inferring the alignment of coordinate systems from user behaviour. Proceedings of the IEEE International Symposium on Mixed and Augmented Reality (ISMAR).

[106] Pucihar K.C., Coulton P., Hutchinson D. Utilizing sensor fusion in markerless mobile augmented reality. Proceedings of the 13th International Conference on Human Computer Interaction with Mobile Devices and Services.

[107] Rahman A.S.M.M., Hossain M.A., Saddik A.E. (2010). Spatial-geometric approach to physical mobile interaction based on accelerometer and IR sensory data fusion. ACM Trans. Multimed. Comput. Commun. Appl..

[108] Grunerbl A., Muaremi A., Osmani V., Bahle G., Ohler S., Troester G., Mayora O., Haring C., Lukowicz P. (2014). Smart-Phone Based Recognition of States and State Changes in Bipolar Disorder Patients. IEEE J Biomed. Health Inf..

[109] Gil G.B., Berlanga de Jesus A., Molina Lopez J.M. inContexto: A fusion architecture to obtain mobile context. Proceedings of the Proceedings of the 14th International Conference on Information Fusion (FUSION).

[110] Kim J., Gracanin D., Quek F. Sensor-fusion walking-in-place interaction technique using mobile devices. Proceedings of the 2012 IEEE Virtual Reality Short Papers and Posters (VRW).

[111] Abadi M.J., Gu Y., Guan X., Wang Y., Hassan M., Chou C.T. (2013). Improving Heading Accuracy in Smartphone-based PDR Systems using Multi-Pedestrian Sensor Fusion. Electr. Eng..

[112] Altini M., Vullers R., Van Hoof C., van Dort M., Amft O. Self-calibration of walking speed estimations using smartphone sensors. Proceedings of the 2014 IEEE International Conference on Pervasive Computing and Communications Workshops (PERCOM Workshops).

[113] Tsai P.H., Lin Y.J., Ou Y.Z., Chu E.T.H., Liu J.W.S. (2014). A Framework for Fusion of Human Sensor and Physical Sensor Data. IEEE Trans. Syst. Man Cybern. Syst..

[114] Lee B.G., Chung W.Y. (2012). A smartphone-based driver safety monitoring system using data fusion. Sensors.

[115] Chen D., Schmidt A., Gellersen H.W. An Architecture for Multi-Sensor Fusion in Mobile Environments. http://www.cs.cmu.edu/datong/Fusion99.pdf.

[116] Sashima A., Ikeda T., Kurumatani K. Toward Mobile Sensor Fusion Platform for Context Aware Services. http://cdn.intechopen.com/pdfs-wm/6799.pdf.

[117] Ayub S., Bahraminisaab A., Honary B. A sensor fusion method for smart phone orientation estimation. Proceedings of the 13th Annual Post Graduate Symposium on the Convergence of Telecommunications, Networking and Broadcasting.

[118] Zhu W., Liu L., Yin S., Hu S., Tang E.Y., Wei S. (2013). Motion-sensor fusion-based gesture recognition and its VLSI architecture design for mobile devices. Int. J. Electron..

[119] van de Ven P., Bourke A., Tavares C., Feld R., Nelson J., Rocha A., O Laighin G. Integration of a suite of sensors in a wireless health sensor platform. Proceedings of the 2009 IEEE Sensors.

[120] Chen J., Low K.H., Tan C.K.Y., Oran A., Jaillet P., Dolan J.M., Sukhatme G.S. (2012). Decentralized Data Fusion and Active Sensing with Mobile Sensors for Modeling and Predicting Spatiotemporal Traffic Phenomena. arXiv preprint arXiv:1206.6230.

[121] Zhao D., Ma H., Tang S. COUPON: Cooperatively Building Sensing Maps in Mobile Opportunistic Networks. Proceedings of the 2013 IEEE 10th International Conference on Mobile Ad-Hoc and Sensor Systems (MASS).

[122] Zhao D., Ma H., Tang S., Li X.Y. (2015). COUPON: A Cooperative Framework for Building Sensing Maps in Mobile Opportunistic Networks. IEEE Trans. Parallel Distrib. Syst..

[123] Deyun G., Tao Z., Dong P., Sidong Z. A general multi-sensor node in wireless sensor networks. Proceedings of the 2009. ICCTA ’09. IEEE International Conference on Communications Technology and Applications.

[124] Fortino G., Galzarano S., Gravina R., Li W. (2015). A framework for collaborative computing and multi-sensor data fusion in body sensor networks. Inf. Fusion.

[125] Zheng E., Chen B., Wang X., Huang Y., Wang Q. (2014). On the Design of a Wearable Multi sensor System for Recognizing Motion Modes and Sit to stand Transition. Int. J. Adv. Robot. Syst..

[126] Chen J.I.Z. (2009). An Algorithm of Mobile Sensors Data Fusion Orientation tracking for Wireless Sensor Networks. Wirel. Pers. Commun..

[127] Saeedi S., Moussa A., El-Sheimy N. (2014). Context-aware personal navigation using embedded sensor fusion in smartphones. Sensors.

[128] Bhuiyan M.Z.H., Kuusniemi H., Chen L., Pei L., Ruotsalainen L., Guinness R., Chen R. (2013). Performance Evaluation of Multi-Sensor Fusion Models in Indoor Navigation. Eur. J. Navig..

[129] Martin H., Bernardos A.M., Tarrio P., Casar J.R. Enhancing activity recognition by fusing inertial and biometric information. Proceedings of the 14th International Conference on Information Fusion.

[130] Bellos C., Papadopoulos A., Rosso R., Fotiadis D.I. (2011). Heterogeneous data fusion and intelligent techniques embedded in a mobile application for real-time chronic disease management. Conf. Proc. IEEE Eng. Med. Biol. Soc..

[131] Martín H., Bernardos A.M., Iglesias J., Casar J.R. (2012). Activity logging using lightweight classification techniques in mobile devices. Pers. Ubiquitous Comput..

[132] Thatte G., Li M., Lee S., Emken B.A., Annavaram M., Narayanan S., Spruijt-Metz D., Mitra U. (2011). Optimal Time-Resource Allocation for Energy-Efficient Physical Activity Detection. IEEE Trans. Signal Process..

[133] Scheuermann B., Ehlers A., Riazy H., Baumann F., Rosenhahn B. Ego-motion compensated face detection on a mobile device. Proceedings of the 2011 IEEE Computer Society Conference on Computer Vision and Pattern Recognition Workshops (CVPRW).

[134] Klingeberg T., Schilling M. (2012). Mobile wearable device for long term monitoring of vital signs. Comput. Methods Progr. Biomed..

[135] Jin Y.Y., Toh H.S., Soh W.S., Wong W.C. A Robust Dead-Reckoning Pedestrian Tracking System with Low Cost Sensors. Proceedings of the 2011 IEEE International Conference on Pervasive Computing and Communications (PerCom).

[136] Grunerbl A., Muaremi A., Osmani V., Bahle G., Ohler S., Troester G., Mayora O., Haring C., Lukowicz P. (2014). Smart-Phone Based Recognition of States and State Changes in Bipolar Disorder Patients. IEEE J. Biomed. Health Inf..

[137] García F., Jiménez F., Anaya J.J., Armingol J.M., Naranjo J.E., de la Escalera A. (2013). Distributed Pedestrian Detection Alerts Based on Data Fusion with Accurate Localization. Sensors.

[138] Ou S., Fagg A.H., Shenoy P., Chen L. (2009). Application Of Reinforcement Learning In Multisensor Fusion Problems With Conflicting Control Objectives. Intell. Autom. Soft Comput..

[139] Wang J., Chen G., Kotz D. A sensor-fusion approach for meeting detection. Proceedings of the Workshop on Context Awareness at the Second International Conference on Mobile Systems, Applications, and Services.

[140] Seoane F., Mohino-Herranz I., Ferreira J., Alvarez L., Buendia R., Ayllón D., Llerena C., Gil-Pita R. (2014). Wearable Biomedical Measurement Systems for Assessment of Mental Stress of Combatants in Real Time. Sensors.

[141] Luque R., Casilari E., Moron M.J., Redondo G. (2014). Comparison and characterization of Android-based fall detection systems. Sensors.

[142] Salah O., Ramadan A.A., Sessa S., Ismail A.A., Fujie M., Takanishi A. (2014). ANFIS-based Sensor Fusion System of Sit- to- stand for Elderly People Assistive Device Protocols. Int. J. Autom. Comput..

[143] Kleinberger T., Becker M., Ras E., Holzinger A., Müller P. Ambient Intelligence in Assisted Living: Enable Elderly People to Handle Future Interfaces. Proceedings of the 4th International Conference on Universal Access in Human-computer Interaction: Ambient Interaction.

[144] Holzinger A., Searle G., Pruckner S., Steinbach-Nordmann S., Kleinberger T., Hirt E., Temnitzer J. Perceived usefulness among elderly people: Experiences and lessons learned during the evaluation of a wrist device. Proceedings of the 2010 4th International Conference on Pervasive Computing Technologies for Healthcare (PervasiveHealth).

[145] Ziefle M., Rocker C., Holzinger A. Medical Technology in Smart Homes: Exploring the User’s Perspective on Privacy, Intimacy and Trust. Proceedings of the 2011 IEEE 35th Annual Computer Software and Applications Conference Workshops (COMPSACW).

[146] Macias E., Suarez A., Lloret J. (2013). Mobile sensing systems. Sensors.

[147] Volkov A.S. (2015). Accuracy bounds of non-Gaussian Bayesian tracking in a NLOS environment. Signal Process..

[148] Gulrez T., Kavakli M. Precision Position Tracking in Virtual Reality Environments using Sensor Networks. Proceedings of the IEEE International Symposium on Industrial Electronics, 2007. ISIE 2007.

[149] Chen C.A., Chen S.L., Huang H.Y., Luo C.H. (2011). An asynchronous multi-sensor micro control unit for wireless body sensor networks (WBSNs). Sensors.

[150] Castro Garrido P., Luque Ruiz I., Gomez-Nieto M.A. AGATHA: Multiagent system for user monitoring. Proceedings of the 2012 IEEE International Conference on Consumer Electronics - Berlin (ICCE-Berlin).

[151] Broggi A., Debattisti S., Panciroli M., Grisleri P., Cardarelli E., Buzzoni M., Versari P. (2011). High performance multi-track recording system for automotive applications. Int. J. Autom. Technol..

[152] Dong J., Zhuang D., Huang Y., Fu J. (2009). Advances in multi-sensor data fusion: algorithms and applications. Sensors.

[153] Garcia N.M. (2016). A Roadmap to the Design of a Personal Digital Life Coach. ICT Innovations.

